# Case Report: Acute hepatitis A virus infection presenting with direct antiglobulin test-negative autoimmune hemolytic anemia and α-thalassemia trait

**DOI:** 10.12688/f1000research.156586.1

**Published:** 2024-10-14

**Authors:** Habiba Debbabi, Eya Chakroun, Hajer Hassine, Hela Kchir, Dhouha Cherif, Haythem Yacoub, Nadia Maamouri

**Affiliations:** 1 Gastroenterology 'B' Department, La Rabta University Hospital Center, Tunis, Tunis, Tunisia; 2 Hematology Department and Blood Bank, La Rabta University Hospital Center, Tunis, Tunis, Tunisia

**Keywords:** DAT-negative, autoimmune hemolytic anemia, hepatitis A infection

## Abstract

Reports from the literature have discussed patients presenting Hepatitis A virus infection with hemolytic anemia, specifically with glucose-6-phosphate dehydrogenase deficiency. However, autoimmune hemolytic anemia (AIHA) has been rarely reported. We present a challenging case of Coombs-negative hemolytic anemia as initial manifestation of hepatitis A virus infection in a silent carrier of α-thalassemia.

## Introduction

The hepatitis A virus (HAV) is the most common type of acute viral hepatitis. It is usually a self-limiting disease with variable clinical presentation. Symptoms can range from mild to severe, and usually include jaundice and digestive signs. Various extrahepatic manifestations can occur with acute hepatitis A infection, such as neurological complications and acute kidney injuries. However, extrahepatic immune complications remain rare. ^1^ Hemolytic anemia has also been observed as a hematological complication associated with HAV infection in several case studies, especially in patients with glucose-6-phosphate dehydrogenase (G6PD) deficiency. ^2, 3^ However, autoimmune hemolytic anemia (AIHA) during the course of HAV infection has rarely been described. ^4– 6^ As the immune nature of anemia is caused by the positivity of the Direct Antiglobulin test (DAT), DAT-negative AIHA cases could evade diagnosis if not recognized. Herein, we present a challenging case of 39 a year-old man with α-thalassemia trait who presented with acute HAV infection revealed by DAT-negative AIHA.

## Case report

A 39-year-old man with no significant past medical history presented with jaundice, and fatigue started 5 days prior to presentation. He had no remarkable family history of hematological disorders. He had received no drugs and had no prior hospitalization. At the time of admission, the patient was severely jaundiced and pale. The body temperature was 37.2°C. pulse rate was 88 beats per minute (bpm). blood pressure was 120/70 mmHg. His abdomen was soft. Hepatomegaly (liver span, 17.5 cm) and splenomegaly (3 cm below the left costal margin) were noted. He had no palpable lymphadenopathy, and the remainder of the physical examination was normal.

Admission laboratory evaluation of the patient was as follows: hemoglobin (Hb): 5.7 g/dL, mean corpuscular volume (MCV): 123 fL, absolute reticulocyte count: 219 400/μL, platelet count: 137 000/μL and white blood cell count: 5820/μL. A peripheral blood smear revealed anisocytosis, polychromatophils, and dacryocytes. The direct antiglobulin test (DAT) using a gel microcolumn (Low Ionic Strength Solution (LISS), Bio-Rad Laboratories) was negative for IgG and C3d. An elevation of serum aspartate aminotransferase (AST) 44.17 IU/L (normal: 5-34); alanine aminotransferase (ALT) 100.23 IU/L (normal: < 55); gammaglutamyltransferase (ɤGT) 276.97 IU/l (normal: 12–64); total bilirubin (TBil) 319 μmol/L (normal: <12) with an indirect fraction of 111 μmol/L and a high LDH level (540 U/L) were noted. Renal function tests, electrolyte levels, prothrombin time, and autoimmune tests were normal.

Further investigations of cholestatic hepatitis showed that the viral serologic studies were positive for anti-HAV IgM antibody and negative for anti-HAV IgG. Tests for hepatitis B virus surface antigen, hepatitis C virus (HCV) antibody (anti-HCV), and screening for HIV, CMV, and EBV were negative.

Abdominal Ultrasonography showed hepatosplenomegaly but no evidence of biliary obstruction. Bili MRI revealed no abnormalities.

Given hemolysis, constitutional causes, including inherited spherocytosis and G6PD deficiency, were excluded. However, hemoglobin electrophoresis revealed an α-thalassemia trait (97.5% hemoglobin A1, 0.9% hemoglobin F, and 1.6% hemoglobin A2). Cell flow cytometry was negative for paroxysmal nocturnal hemoglobinuria.

Furthermore, a remarkably high serum ferritin level (512μg/L). folate level was low (2.5 μg/L), but vitamin B12 level was normal. Thyroid function, Anti-intrinsic factor (IF) antibodies, and anti-parietal cell antibodies were also negative. DAT was repeated during hospitalization and performed with monospecific anti-human globulin (AHG) reagents, including anti-IgG, -IgA, -IgM, -C3c, and -C3d antisera, and yielded negative results. The eluate was negative.

Based on clinical and laboratory findings, the patient was diagnosed with acute HAV infection complicated by DAT-negative AIHA. Bone marrow biopsy was normal, with no evidence of lymphoproliferative disorder.

The patient symptoms improved and remained stable during hospitalization. The patient’s blood cell count showed a slight upward trend. The Hb level reached 6.8 g/dL on the 10 ^th^ day of hospital stay. TBil levels decreased to 101 μmol/L and liver enzyme levels showed a marked reduction. The patient was discharged in good condition two weeks after admission and was jointly cared for in the Hematological outpatient department.

It is important to note that 3 months after the acute episode, the patient had recurrence of his symptoms with re-elevation of the serum TBil level (382 μmol/L), and the Hb level began to drop to 6.2 mg/dL. The persistence of hepatitis A IgM antibodies prompted the consideration of relapsing hepatitis A. Only by the 5 ^th^ month of follow-up, hepatitis A IgM antibodies disappeared, and oral prednisone therapy (70 mg, 1.5 mg/kg/day) was started; Hb Levels rapidly increased to 7.1 g/dL **.** Corticosteroids were maintained at the same dose for 6 weeks and then tapered gradually over several weeks. The patient’s condition improved within the first month of steroid therapy, and the hemoglobin level reached 10.1 g/dL. During the follow-up, the CBC was completely re-normalized.

## Discussion

Autoimmune hemolytic anemia has been reported in association with various hepatotropic viruses, notably Epstein-Barr virus, cytomegalovirus, and hepatitis B. A well-established link exists between chronic active hepatitis and AIHA. We present a rare case of acute hepatitis A, manifesting as a direct antiglobulin test (DAT)-negative AIHA.

In advanced liver disease, reduced hemoglobin levels are indicative of poor outcomes, such as liver decompensation and the onset of acute-on-chronic liver failure. ^7, 8^

Anemia can have several etiologies (acute or chronic blood loss, hemolysis, and malabsorption). A combination of AIHA and viral hepatitis has been reported in the literature, and Hepatitis E virus, hepatitis C virus chronic infection, cytomegalovirus infection, and HAV acute hepatitis are epidemiologically associated with AIHA. ^6^

However, the pathogenic mechanisms underlying hemolysis in acute hepatitis remain unknown and have not been completely elucidated. ^9– 11^ The presentation of HAV infection varies from complete lack of symptoms to acute/fulminant hepatitis, but gastrointestinal symptoms, fever, and malaise are frequent. ^1^ In this condition, the diagnosis of concomitant AIHA may be challenging, as common signs of AIHA, such as fatigue, jaundice, and pallor, may go undetected in patients with a severe presentation. Indeed, AIHA has been reported during the course of HAV infection. ^12– 14^ The hypothesis that hemolysis is induced by circulating antibodies or the effect of the virus on red blood cells has been proposed by several authors. ^12, 15, 16^ To date, only two studies have detected autoantibodies that were IgM antibodies against triosephosphate isomerase (anti-TPI). ^12, 13^

Antibodies against TPI damage enzyme activity and reduce the osmotic resistance of erythrocytes. The presence of these antibodies is hypothesized to be linked to the reactivation of latent persistent Epstein-Barr virus (EBV) infection. ^13^ However, in our patient, EBV serology was negative. No anti-TPI antibody was detected, as reported in other studies.

The occurrence of contributing factors seems to be necessary, as red cell survival in the absence of an underlying red cell abnormality can be shortened by acute infectious hepatitis, but the rate of destruction of RBCs due to the effect of the virus alone is insufficient to induce hemolytic anemia. ^2, 3, 16– 18^

The hemolytic anemia can increase up to 70–87% in patients with G6PD deficiency complicated by acute hepatitis, ^19^ which may result in a severe clinical presentation. ^2, 3, 15, 20, 21^

The mechanism of hemolysis in these cases could result from the accumulation of oxidants due to hepatic dysfunction, which leads to reduced glutathione levels and induces hemolysis. ^3, 9^ G6PD deficiency was not found in our patient, but he carried the α-thalassemia trait.

It is worth to note that there is a high incidence of thalassemia (2.5-25%) in the tropical subtropical regions of Africa, the Asian subcontinent, and Southeast Asia, the Middle East, and particularly in the Mediterranean basin where milder forms of the disease are frequently observed. ^22^ Silent carriers of α-thalassemia are typically asymptomatic and may exhibit either a normal blood count or mild microcytic hypochromic anemia; therefore, no specific treatment is recommended for these patients. ^23^ The involvement of this inherited blood disorder in the onset of hemolysis in cases of acute hepatitis A remains unknown as no similar reports have been reported in the literature. However, it seems unlikely that hemolysis is related to the α-thalassemia trait because of the absence of previous hemolytic episodes.

Hemolysis may also be an autoimmune process. Positive DAT is a diagnostic hallmark of AIHA. However, negative DAT may be found in some cases, and it is considered a challenging situation because diagnosis and management may be delayed. ^6^

The incidence of DAT-negative AIHA is approximately 3 to 11% in all cases. ^24, 25^ The main causes of negative DAT could be a low level of antibodies on the RBCs, a lower sensitivity of DAT, or an autoantibody type IgA or IgM, which many commercial DAT reagents could miss because they only contain anti-IgG and anti-C3. ^26, 27^

Subsequent methods have been developed to achieve greater sensitivity in detecting red cell sensitization by IgG below the threshold of the routine commercial DAT or by IgA alone, or rarely (monomeric) IgM alone (Eluate, test with anti-IgA or anti-IgM antisera antiglobulin test, enzyme-linked anti-IgG assay, and flow cytometry to detect red cell IgG). ^28^ It is important to note that these tests have a low predictive value, and thus, the test results should be interpreted according to clinical and biological data for DAT-negative AIHA. ^27^

In our patient, both DAT monospecific AHG reagents, including anti-IgG, -IgA, -IgM, -C3c, -C3d anti-sera, and eluate, were performed and yielded negative results. Similarly, the lack of an alternative confirmatory test should not delay treatment. Thus, diagnostic assessment must rule out all hereditary and acquired causes of hemolytic anemia. In fact, the conjunction of hemolysis, denial of other hemolytic diseases, and responsiveness to steroid treatments are key to establishing a diagnosis of DAT-negative AIHA. ^29^ In fact, corticosteroids are the cornerstone of AIHA treatment; splenectomy and rituximab are the second-line treatments. Transfusion is required in cases of severe anemia. ^30^

As no drugs are targeted for the treatment of acute hepatitis A infection, patients are currently treated symptomatically with intravenous fluids and antipyretics as indicated. ^30^

The most common hemolytic disorders associated with viral hepatitis are brief. However, in some patients, hemolysis persists for longer intervals even after recovery from viral hepatitis. ^31^ In addition, the length of time that the IgM anti-HAV test remains positive varies widely, from 4 to 32 months in some studies. ^32, 33^

In our patient, the anemia persisted over five months after the onset of jaundice, and we hypothesized that this hemolytic state could not be related to the severity of liver disease, but rather suggests that an immunological disorder linked to HAV could exist and persist even after infectious hepatitis.

## Conclusion

Hepatitis A is common worldwide, with a wide range of manifestations that can evade the diagnosis of concomitant hemolytic anemia. This case highlights the importance of recognizing the occurrence of AIHA, even if DAT is negative. Therefore, extensive medical and laboratory workup may be warranted, and responsiveness to steroid treatment is considered a supportive element of DAT-negative AIHA. However, the involvement of the alpha-thalassemia trait in our patient remains unclear.

## Consent

Written informed consent for publication of his clinical details was obtained from the patient.

## Data Availability

No data are associated with this article.
