# Case Report: Acute hepatitis A virus infection presenting with direct antiglobulin test-negative autoimmune hemolytic anemia and α-thalassemia trait

**DOI:** 10.12688/f1000research.156586.2

**Published:** 2025-04-15

**Authors:** Habiba Debbabi, Eya Chakroun, Hajer Hassine, Hela Kchir, Dhouha Cherif, Haythem Yacoub, Nadia Maamouri

**Affiliations:** 1 Gastroenterology 'B' Department, La Rabta University Hospital Center, Tunis, Tunis, Tunisia; 2 Hematology Department and Blood Bank, La Rabta University Hospital Center, Tunis, Tunis, Tunisia

**Keywords:** DAT-negative, autoimmune hemolytic anemia, hepatitis A infection

## Abstract

Reports from the literature have discussed patients presenting Hepatitis A virus infection with hemolytic anemia, specifically with glucose-6-phosphate dehydrogenase deficiency. However, autoimmune hemolytic anemia (AIHA) has been rarely reported. We present a challenging case of Coombs-negative hemolytic anemia as initial manifestation of hepatitis A virus infection in a silent carrier of α-thalassemia.

## Introduction

Hepatitis A virus (HAV) is the most common type of acute viral hepatitis. It is usually a self-limiting disease with variable clinical presentation. Symptoms can range from mild to severe and usually include jaundice and digestive signs. Various extrahepatic manifestations can occur with acute hepatitis A infection, such as neurological complications and acute kidney injuries. However, extrahepatic immune complications are uncommon. ^1^ Hemolytic anemia has also been reported as a hematological complication associated with HAV infection in several case studies, particularly in patients with glucose-6-phosphate dehydrogenase (G6PD) deficiency. ^2^ ^,^ ^3^ In contrast, autoimmune hemolytic anemia (AIHA) occurring during the course of HAV infection has been rarely documented in the literature. ^4^ ^–^ ^6^ Given that the immune-mediated nature of anemia is typically confirmed by a positive Direct Antiglobulin test (DAT), cases of DAT-negative AIHA may go undiagnosed if not recognized. Herein, we present a challenging case of a 39-year-old man with α-thalassemia trait who presented with acute HAV infection revealed by DAT-negative AIHA.

## Case report

A 39-year-old man with no significant past medical history presented with jaundice and fatigue that began five days prior to admission. He had no notable family history of hematological disorders, no prior hospitalizations, and no history of drug use. On examination, the patient appeared severely jaundiced and pale. His vital signs were as follows: body temperature, 37.2°C; pulse rate, 88 beats per minute (bpm); and blood pressure, 120/70 mmHg. Abdomen examination revealed soft abdomen with hepatomegaly (liver span, 17.5 cm) and splenomegaly (3 cm below the left costal margin). There was no palpable lymphadenopathy, and the remainder of the physical examination was unremarkable.

The patient admission laboratory evaluation revealed severe anemia with hemoglobin (Hb) level of 5.7 g/dL and macrocytosis, as indicated by a mean corpuscular volume (MCV): 123 fL, absolute reticulocyte count was 219 400/μL, platelet count: 137 000/μL and white blood cell count of 5820/μL. A peripheral blood smear analysis demonstrated anisocytosis, polychromatophils, and dacryocytes. The direct antiglobulin test (DAT) using a gel microcolumn (Low Ionic Strength Solution (LISS), Bio-Rad Laboratories) was negative for both IgG and C3d.

Biochemical analysis showed elevated liver enzymes, with aspartate aminotransferase (AST) at 44.17 IU/L (reference range: 5-34 IU/L), alanine aminotransferase (ALT) at 100.23 IU/L (reference range: < 55 IU/L, and gamma-glutamyltransferase (ɤGT) at 276.97 IU/l (reference range: 12–64 IU/L). Additionally, there was significant hyperbilirubinemia, with a total bilirubin (TBil) of 319 μmol/L (reference range: <17 μmol/L), including an indirect fraction of 111 μmol/L. While lactate dehydrogenase (LDH) was markedly elevated at 540 U/L (reference range: 125-245 U/L), haptoglobin testing was not available due to resource limitations. Renal function tests, electrolyte levels, prothrombin time, and autoimmune screenings were within normal limits.

Further investigations of cholestatic hepatitis revealed positive serologic findings for anti-HAV IgM antibodies, while anti-HAV IgG antibodies were negative. Screening for other viral etiologies, including hepatitis B surface antigen (HBsAg), hepatitis B core antibodies (anti-HBc), hepatitis C virus (HCV) antibodies), as well as HIV, cytomegalovirus (CMV), and Epstein-Barr virus (EBV), yielded negative results.

Abdominal ultrasonography demonstrated hepatosplenomegaly without evidence of biliary tract obstruction. Biliary Magnetic Resonance Imaging revealed no abnormalities.

Given the presence of hemolysis, constitutional causes were ruled out. Hereditary spherocytosis was excluded based on a normal erythrocyte osmotic fragility test (OFT). The Eosin-5′-maleimide (EMA) binding test (EMABT) and ektacytometry could not be performed due to limited resources. G6PD deficiency was also ruled out through G6PD level determination, notably, there was no family history suggestive of inherited hemolytic anemia in either case. On the other hand, capillary hemoglobin electrophoresis revealed an α-thalassemia trait (97.5% hemoglobin A1, 0.9% hemoglobin F, and 1.6% hemoglobin A2). Cell flow cytometry was negative for paroxysmal nocturnal hemoglobinuria.

Furthermore, laboratory investigations revealed a remarkably elevated serum ferritin level of 512 μg/L. The folate level was low at 2.5 μg/L, while the vitamin B12 level remained within the normal range. Additional evaluations, including thyroid function, anti-intrinsic factor (IF) antibodies, and anti-parietal cell antibodies, were all negative. DAT was repeated during hospitalization and performed with monospecific anti-human globulin (AHG) reagents, including anti-IgG, -IgA, -IgM, -C3c, and -C3d antisera, and yielded negative results. The eluate was negative.

Based on clinical and laboratory findings, the patient was diagnosed with acute HAV infection complicated by DAT-negative AIHA. Bone marrow biopsy was normal, with no evidence of lymphoproliferative disorder.

Vitamin B9 supplementation was initiated, and the patient’s symptoms improved. The blood cell count exhibited a slight upward trend, with Hb reaching 6.8 g/dL and platelet levels returning to normal by the tenth day of hospitalization. TBil levels decreased to 101 μmol/L, and liver enzyme levels showed a marked reduction. The patient was discharged in stable condition two weeks after admission and continued to receive follow-up care in the Hematological outpatient department.

Notably, three months after the acute episode, the patient experienced a recurrence of symptoms accompanied by a rise in serum TBil level to 382 μmol/L and a decline in Hb to 6.2 mg/dL. The persistent presence of hepatitis A IgM antibodies prompted the consideration of relapsing hepatitis A. Oral prednisone therapy was initiated at 1.5 mg/kg/day, resulting in rapid Hb increase to 7.1 g/dL within few days. Prednisone was continued at the initial dose for six weeks before tapering. The patient’s condition improved within the first month of steroid therapy, and the hemoglobin level reached 10.1 g/dL. Hematologic response was observed within four weeks (Hb 10.1 g/dL), with full CBC normalization by follow-up.

## Discussion

Autoimmune hemolytic anemia has been reported in association with various hepatotropic viruses, notably EBV, CMV, and hepatitis B. While chronic active hepatitis has a well-documented association with AIHA, we report a rare case of acute HAV infection presenting as a DAT-negative AIHA.

Anemia can have several etiologies (acute or chronic blood loss, hemolysis, and malabsorption). A combination of AIHA and viral hepatitis has been reported in the literature, and Hepatitis E virus, hepatitis C virus chronic infection, CMV infection, and HAV infection are epidemiologically associated with AIHA. ^6^

Nevertheless, the pathogenic mechanisms underlying hemolysis in acute hepatitis remain unknown and have not been completely elucidated. ^7^ ^–^ ^9^ The presentation of HAV infection varies from complete lack of symptoms to acute and even fulminant hepatitis, but gastrointestinal symptoms, fever, and malaise are frequent. ^1^ In this condition, the diagnosis of concomitant AIHA may be challenging, as common signs of AIHA, such as fatigue, jaundice, and pallor, may go undetected in patients with severe presentation. Indeed, AIHA has been reported during the course of HAV infection. ^10^ ^–^ ^12^ The hypothesis that hemolysis is induced by circulating antibodies or the effect of the virus on red blood cells has been proposed by several authors. ^10^ ^,^ ^13^ ^,^ ^14^ To date, only two studies have detected autoantibodies that were IgM antibodies against triosephosphate isomerase (anti-TPI). ^10^ ^,^ ^11^

Anti-TPI antibodies impair enzyme function and decrease erythrocyte osmotic resistance. The presence of these antibodies is hypothesized to be linked to the reactivation of latent persistent EBV infection. ^11^ In our patient, EBV serology was negative, and no anti-TPI antibodies were detected. The presence of contributing factors seems essential, as acute infectious hepatitis can shorten red cell survival even without an underlying red cell abnormality. However, the virus alone is generally insufficient to cause hemolytic anemia due to its limited impact on red blood cell destruction ^2^ ^,^ ^3^ ^,^ ^14^ ^–^ ^16^ Hemolytic anemia can increase to 70–87% in patients with G6PD deficiency complicated by acute hepatitis, ^17^ potentially leading to a severe clinical presentation. ^2^ ^,^ ^3^ ^,^ ^13^ ^,^ ^18^ ^,^ ^19^ In these cases, hemolysis may result from the accumulation of oxidants due to hepatic dysfunction, leading to decreased glutathione levels and subsequent hemolysis. ^3^ ^,^ ^7^ Although our patient did not have G6PD deficiency, he was found to carry the α-thalassemia trait.

It is worth noting that thalassemia has a high incidence (2.5-25%) in the tropical and subtropical regions of Africa, the Asian subcontinent, Southeast Asia, and the Middle East. The Mediterranean basin, in particular, exhibits a high prevalence, with milder forms of the disease frequently observed. ^20^ Silent carriers of α-thalassemia are typically asymptomatic and may exhibit either a normal blood count or mild microcytic hypochromic anemia. Therefore, no specific treatment is required for these patients. ^21^ The role of this inherited blood disorder in triggering hemolysis during acute hepatitis A remains unclear, as no similar cases have been documented in the literature. However, hemolysis is unlikely to be linked to the α-thalassemia trait, given the absence of previous hemolytic episodes.

Hemolysis may also be an autoimmune process. Positive DAT is a diagnostic hallmark of AIHA. But negative DAT may be found in some cases, and it is considered a challenging situation because diagnosis and management may be delayed. ^6^ The incidence of DAT-negative AIHA is approximately 3 to 11% in all cases. ^22^ ^,^ ^23^ The main causes of negative DAT could be a low level of antibodies on the RBCs, a lower sensitivity of DAT, or an autoantibody type IgA or IgM, which many commercial DAT reagents could miss because they only contain anti-IgG and anti-C3. ^24^ ^,^ ^25^

Subsequent methods have been developed to enhance sensitivity in detecting red cell sensitization by IgG below the threshold of the routine commercial DAT, as well as by IgA alone, or rarely, monomeric IgM alone. These include eluate testing, antiglobulin tests with anti-IgA or anti-IgM antisera, enzyme-linked anti-IgG assays, and flow cytometry for red cell IgG detection. ^26^ However, it is important to note that these tests have a low predictive value, therefore, results should be interpreted in conjunction with clinical and biological data for DAT-negative AIHA. ^25^

In our patient, both DAT monospecific AHG reagents, including anti-IgG, -IgA, -IgM, -C3c, -C3d anti-sera, and eluate, were performed and yielded negative results. Similarly, the lack of an alternative confirmatory test should not delay treatment. Thus, diagnostic assessment must rule out all hereditary and acquired causes of hemolytic anemia. In fact, the combination of hemolysis, the exclusion of other hemolytic diseases, and positive response to steroid therapy are critical factors in establishing a diagnosis of DAT-negative AIHA, ^27^ as demonstrated in our case. Corticosteroids remain the cornerstone of AIHA treatment, while splenectomy and rituximab serve as second-line therapeutic options. Transfusion is required in cases of severe anemia. ^28^

Since there are no targeted drugs for the treatment of acute hepatitis A infection, patients are managed symptomatically with intravenous fluids and antipyretics, as indicated. ^28^ The most common hemolytic disorders associated with viral hepatitis are brief. However, in some patients, hemolysis persists for longer intervals, even after recovery from viral hepatitis. ^29^ In addition, the length of time that the IgM anti-HAV test remains positive varies widely, from 4 to 32 months in some studies. ^30^ ^,^ ^31^

In our patient, the anemia persisted over five months after the onset of jaundice, and we hypothesized that this hemolytic state could not be related to the severity of liver disease but rather suggests that an immunological disorder linked to HAV could exist and persist even after infectious hepatitis.

## Conclusion

Hepatitis A is prevalent worldwide and presents with a wide spectrum of manifestations, which can obscure the diagnosis of concomitant hemolytic anemia. This case highlights the need to consider AIHA, even in the absence of DAT. A thorough medical and laboratory evaluation may be necessary, and a positive response to steroid treatment can serve as a supportive indicator of DAT-negative AIHA. However, the role of the alpha-thalassemia trait in our patient remains unclear.

## Consent

Written informed consent for publication of his clinical details was obtained from the patient.

## Data Availability

No data are associated with this article.
