# Supplementary material for: Case Report: Acute hepatitis A virus infection presenting with direct antiglobulin test-negative autoimmune hemolytic anemia and α-thalassemia trait
Source: F1000Res. 2025 Apr 15;13:1224. Originally published 2024 Oct 14. [Version 2] doi: 10.12688/f1000research.156586.2 (PMC12120424; doi:10.12688/f1000research.156586.2)
Supplement: Peer review history

# Reviewer report: Reviewer response for version 2

**DOI:** 10.5256/f1000research.180216.r379388

**Published:** 2025-05-29

**Authors:** Mhd Kutaiba Albuni

**Affiliations:** 1 TriHealth Good Samaritan Hospital, Cincinnati, Ohio, USA

This case report discusses a 39-year-old male with acute hepatitis A virus (HAV) infection presenting with direct antiglobulin test (DAT)-negative autoimmune hemolytic anemia (AIHA) and α-thalassemia trait. Despite the negative DAT, the patient was diagnosed with AIHA based on clinical and laboratory findings, including elevated liver enzymes, jaundice, and elevated lactate dehydrogenase (LDH) levels. The patient responded positively to corticosteroid treatment, which further supported the diagnosis of DAT-negative AIHA. This case is noteworthy for highlighting the rare presentation of AIHA in the context of HAV infection and α-thalassemia trait, as well as the diagnostic challenges posed by DAT-negative cases.

 This case report offers a valuable contribution to the literature on the rare presentation of DAT-negative AIHA in HAV infection. However, to strengthen the case and improve its scientific rigor, the authors should consider addressing the following:

- Provide more detailed explanations for the exclusion of other causes of hemolysis.
- Clarify the role of α-thalassemia trait in the pathogenesis of AIHA in this patient.
- Expand on the molecular mimicry hypothesis and explore potential novel HAV variants.
- Include more detailed follow-up data and clarify the rationale behind the initiation of steroid therapy.

Are enough details provided of any physical examination and diagnostic tests, treatment given and outcomes?

Yes

Is the case presented with sufficient detail to be useful for other practitioners?

Yes

Is sufficient discussion included of the importance of the findings and their relevance to future understanding of disease processes, diagnosis or treatment?

Yes

Is the background of the case’s history and progression described in sufficient detail?

Yes

Reviewer Expertise:

Hepatitis

I confirm that I have read this submission and believe that I have an appropriate level of expertise to confirm that it is of an acceptable scientific standard.

# Reviewer report: Reviewer response for version 1

**DOI:** 10.5256/f1000research.171914.r336132

**Published:** 2024-11-19

**Authors:** Shiv Sekhar Chatterjee

**Affiliations:** 1 All India Institute of Medical Sciences, Kalyani, WB, India

1. What is Bili MRI? Please explain in full form.

 2. Please include a figure of the peripheral blood film of this patient.

 3. Please state how spherocytosis and G6PD deficiency were excluded.

 4. Also, state what finding on flow cytometry helped authors rule out paroxysmal nocturnal hemoglobinuria. Please include the flow cytometry graphical read out as a picture.

 5. What was done for the low folate levels of this patient? 

 6. Why was Serum Haptoglobin not measured in this patient?

 7. The Platelet count in this patient was relatively low at 137000/microliter. Was Evan's syndrome a differential diagnosis? 

 8. Is there any significance of the high serum ferritin levels in this patient? Kindly mention in discussion. 

 9. Why do you consider this patient to be a case of DAT negative AIHA? Which diagnostic criteria are fulfilled? Please mention in your Discussion. 

 10. How do authors conclude that the patient was suffering from Alpha Thalassemia Trait? Is it only from hemoglobin electrophoresis? Which electrophoresis was done? HPLC or capillary electrophoresis? Was PCR done for the diagnosis of Alpha Thalassemia trait?

Are enough details provided of any physical examination and diagnostic tests, treatment given and outcomes?

Partly

Is the case presented with sufficient detail to be useful for other practitioners?

Yes

Is sufficient discussion included of the importance of the findings and their relevance to future understanding of disease processes, diagnosis or treatment?

Partly

Is the background of the case’s history and progression described in sufficient detail?

Yes

Reviewer Expertise:

Microbiology, Laboratory Hematology only

I confirm that I have read this submission and believe that I have an appropriate level of expertise to confirm that it is of an acceptable scientific standard, however I have significant reservations, as outlined above.

# Response

**Published:** 2025-01-22

**Authors:** Hajer Hassine

**Affiliations:** Gastroenterology B, La Rabta University Hospital Center, Tunis, Tunis, Tunisia

1. Biliary Magnetic Resonance Imaging (MRI)

 3. For spherocytosis: since peripheral red blood cell morphology examinations, are not sufficiently sensitive or specific. An erythrocyte osmotic fragility test (OFT) was conducted. Unfortunately, Eosin‐5'‐maleimide (EMA) binding test (EMABT) and ektacytometry are not carried out here.

  Howerver, G6PD deficiency was excluded by G6PD level determination.

 For both,  there was no family  history of inherited hemolytic anemia.

 4. Flow cytometry is performed in the hematology laboratory of the Pasteur Institute of Tunis. We do not have the acquisitions of the graphs when the search for a deficit in GPI-anchored molecules is negative.

 5. During hemolysis, folates decrease. It is true that vitamin B9 therapy was prescribed but the patient was not perfectly compliant.

 6. Unfortunately, haptoglobin is a parameter that is not always available in our hospital.

 7. Other platelet counts were normal during follow-up and well before the initiation of corticosteroid therapy in this patient. Therefore, Evans syndrome was not considered as a differential diagnosis.

 8. Hepatic cytolysis could explain the hyperferritinemia which remains < 1000 μg/L

 9. As discussed, we have conducted almost exhaustive investigations to document this hemolysis. It is accepted in the literature that responsiveness to steroid treatments is considered as a convincing argument in favor of the autoimmune mechanism of hemolysis and a key to establishing a diagnosis of DAT-negative AIHA.

 10. capillary electrophoresis was performed.

 PCR is not systematically done for the diagnosis of Alpha Thalassemia trait here.

 Thank you for the review.

# Reviewer report: Reviewer response for version 1

**DOI:** 10.5256/f1000research.171914.r332449

**Published:** 2024-10-30

**Authors:** Rosy Sultana, Kaiissar Mannoor

**Affiliations:** 1 Bangladesh University of Health Sciences,, Dhaka, Bangladesh; 2 Biomedical Engineering, Military Institute of Science and Technology, Dhaka, Dhaka Division, Bangladesh

The case report detailed here is really interesting and deserve attention. The patient had alfa-thalassemia trait. However, carriers of alfa trait have not been demonstrated to cause anemia. Hepatitis A virus (HAV) induced acute hepatitis has been reported to be epidemiologically associated with AIHA without pathogenic mechanisms of underlying hemolysis. There might be several reasons for DAT-negative results, such as-

 i) low sensitivity of the reagents which were used for DAT. 

 ii) low affinity of the IgG antibody

 Chemiluminescence immunoassay (CLIA) based method can be thought to increase the sensitivity of the assays. Diagnostic evaluation performed by the researchers to explore AIHA was elaborate and specific. Since all the test parameters were not in favor of a direct antiglobulin test-positive AIHA, patient's response to steroid treatment was found to be indicative for a diagnosis of DAT-negative AIHA.

 Further approach can be made considering the following issue:

  If the causative HAV is an unconventional mutant variant, it might produce epitope(s) which might be different from that of globally circulating conventional variant. The epitope produced by unconventional mutant variant may mimic erythrocyte epitope. That is, the monoclonal antibody produced in response to causative mutant variant may recognize epitope on erythrocytes causing hemolysis through molecular mimicry.  Usually the binding through molecular mimicry is weak. However, sequencing of immunodominant region of HAV  antigen can be recommended.

Are enough details provided of any physical examination and diagnostic tests, treatment given and outcomes?

Yes

Is the case presented with sufficient detail to be useful for other practitioners?

Yes

Is sufficient discussion included of the importance of the findings and their relevance to future understanding of disease processes, diagnosis or treatment?

Yes

Is the background of the case’s history and progression described in sufficient detail?

Partly

Reviewer Expertise:

Clinical immunology and virology: Hepatitis E virus in pregnancy, SARS-CoV-2 antibodies in diabetic people and healthcare professionals, Association of rheumatoid arthritis with autoimmune thyroid disease

We confirm that we have read this submission and believe that we have an appropriate level of expertise to confirm that it is of an acceptable scientific standard.

# Response

**Published:** 2025-01-22

**Authors:** Hajer Hassine

**Affiliations:** Gastroenterology B, La Rabta University Hospital Center, Tunis, Tunis, Tunisia

As discussed, we have conducted almost exhaustive investigations to document this hemolysis. However, the use of sequencing of the immunodominant region of the HAV antigen had not been considered.

  It is accepted in the literature that  responsiveness to steroid treatments is considered as a convincing argument in favor of the autoimmune mechanism of hemolysis and a key to establishing a diagnosis of DAT-negative AIHA.

 Thank you for the review.
